# Zebra or quagga mussel dominance depends on trade-offs between growth and defense—Field support from Onondaga Lake, NY

**DOI:** 10.1371/journal.pone.0235387

**Published:** 2020-06-29

**Authors:** Lars G. Rudstam, Christopher J. Gandino

**Affiliations:** 1 Department of Natural Resources, Cornell Biological Field Station, Cornell University, Bridgeport, New York, United States of America; 2 Department of Water Environment Protection, Onondaga County, West Syracuse, New York, United States of America; Uppsala Universitet, SWEDEN

## Abstract

Two invasive mussels (zebra mussel, *Dreissena polymorpha* and quagga mussel *D*. *rostriformis bugensis*) have restructured the benthic habitat of many water bodies in both Europe and North America. Quagga mussels dominate in most lakes where they co-occur even though zebra mussels typically invade lakes first. A reversal to zebra mussel over time has rarely been observed. Laboratory experiments have shown that quagga mussels grow faster than zebra mussels when predator kairomones are present and this faster growth is associated with lower investment in anti-predator response in quagga mussels than zebra mussels. This led to the hypothesis that the dominance of quagga mussels is due to faster growth that is not offset by higher vulnerability to predators when predation rates are low, as may be expected in newly colonized lakes. It follows that in lakes with high predation pressure, the anti-predatory investments of zebra mussels should be more advantageous and zebra mussels should be the more abundant of the two species. In Onondaga Lake, NY, a meso-eutrophic lake with annual mussel surveys from 2005 to 2018, quagga mussels increased from less than 6% of the combined mussel biomass in 2007 to 82% in 2009 (from 3 to 69% by number), rates typical of this displacement process elsewhere, but then declined again to 11–20% of the mussel biomass in 2016–2018. Average total mussel biomass also declined from 344–524 g shell-on dry weight (SODW)/m^2^ in 2009–2011 to 34–73 g SODW/m^2^ in 2016–2018, mainly due to fewer quagga mussels. This decline in total mussel biomass and a return to zebra mussel as the most abundant species occurred as the round goby (*Neogobius melanostomus*) increased in abundance. Both the increase to dominance of quagga mussels and the subsequent decline following the increase in this molluscivorous fish are consistent with the differences in the trade-off between investment in growth and investment in defenses of the two species. We predict that similar changes in dreissenid mussel populations will occur in other lakes following round goby invasions, at least on the habitats colonized by both species.

## Introduction

Dreissenid mussels, both zebra mussel (*Dreissena polymorpha*) and quagga mussel (*Dreissena rostriformis bugensis*), are invasive ecosystem engineers with large effects on aquatic ecosystems through filtering and alteration of the benthic habitat (reviews in [1–4]). Both species arrived to North America and Lake Erie in the mid-1980s; zebra mussels were confirmed present in 1986 and quagga mussels in 1989 [5–7]. Zebra mussels then spread rapidly and by 1993 were common across the Laurentian Great Lakes and in many inland lakes [8]. Quagga mussels spread more slowly, but had reached Lake Ontario in 1990, the Mississippi and Ohio Rivers in 1995, lakes Michigan and Huron in 1997, and the Hudson River in 2005 [8, 9]. In addition, it takes longer for quagga mussels to reach maximum abundance after the initial colonization of a lake (average of 12.2 years for quagga mussels versus 2.5 years for zebra mussels [10]. Even so, quagga mussel do end up as the dominant of the two species in most lakes [11–15] and can increase from low densities to the dominant species in two to three years [15, 16]. The displacement of zebra mussels by quagga mussels may increase the effects of these ecosystem engineers if lake-wide dreissenid mussel biomass increases after the quagga mussel becomes the dominant species [17, 18].

There are several physiological and behavioral differences between the two species that may explain the dominance of quagga mussels [10]. Compared to zebra mussels, quagga mussels have a lower metabolic rate, are more resistant to starvation, can grow and reproduce at lower temperatures, and can colonize soft substrata [19–22]. Quagga mussels can therefore build up dense populations on deep, cold bottoms that zebra mussels cannot colonize. This also allows quagga mussels to produce a larger number of veligers, giving them an advantage over zebra mussels in the lottery for settling space [10, 23]. Further, quagga mussels grow better than zebra mussels at low food concentrations [19], thereby having a competitive advantage when dreissenids decrease phytoplankton abundance [7, 24, 25]. In addition, quagga mussels may have higher filtering rates, but investigations of filtering rates that directly compared the two species are inconclusive, with reports of higher filtering rates by quagga mussels [26], higher filtering rates by zebra mussels [19] and no differences [27, 28].

Selective predation cannot be the direct cause for the displacement of zebra mussels by quagga mussels as quagga mussels are more vulnerable to predation because of their thinner shells, less aggregation behavior, lower propensity to seek refuges, and lower attachment strength [29–35]. However, these anti-predation adaptations have a cost. In a series of papers, Naddafi and Rudstam [31–33] explored the difference in anti-predatory investments by the two mussel species, and the consequences of these difference to mussel growth. They compared mussels of both species with and without exposure to predator kairomones. With predator cues present, zebra mussels invested more in shell growth and byssal thread production as well as lowered their filtering rates resulting in lower growth rates compared to quagga mussels that had a more limited response to the predators. These morphological and behavioral responses to predators resulted in lower vulnerability to predation for zebra mussels compared to quagga mussels and both round goby and rusty crayfish (*Orconectes rusticus*) preferred quagga mussels over zebra mussels. Greater investments in anti-predator behavior and morphology by zebra mussels than by quagga mussels have been observed repeatedly in laboratory experiments elsewhere [29, 30, 34, 35].

Although greater investment in anti-predatory adaptations may be an advantage in high predation environments, the additional cost of these investments can be a disadvantage when predation mortality is low. Low predation rates may be expected in newly invaded environments where the predators are not adapted to feeding on mussels, or not yet discovered this new food resource (the enemy release hypothesis of invasion success—[36]). Therefore, Naddafi and Rudstam [32] hypothesized that quagga mussel dominate in many systems because quagga mussel has a more optimal trade-off between resource allocation to growth and to defense than zebra mussels when predation pressure is low, resulting in faster quagga mussel growth rates. This hypothesis (hereafter the trade-off hypothesis) would help explain why quagga mussels dominate also in productive lakes where food limitation is less important and where the deep cold water bottoms are often anoxic. In such lakes, a faster growth rates of quagga mussels in low food environments and cold temperatures should be less important. If the trade-off hypothesis is important, quagga mussels should dominate in lakes with low predation pressure and zebra mussels should dominate in lakes with high predation pressure, such as expected after the arrival of the mussel specialist round goby (*Neogobius melanostomus*), an invasive fish species native to the Ponto-Caspian region that is spreading through North America and Europe [37].

The trade-off hypothesis could be tested against field data from a productive lake that includes both years with high and years with low densities of mussel predators. Herein, we analyze such a data series–a 14 year data set (2005–2018) from Onondaga Lake, New York, USA. This data set consist of annual surveys conducted during years when quagga mussels increased in abundance and during the eight years after the arrival of the round goby in 2010. In addition, the Onondaga Lake data includes information on other aspects of the ecosystem (phytoplankton, zooplankton, fish, nutrients) that can be used to evaluate alternative explanations for changes in mussel abundance [38]. Based on our trade-off hypothesis, we expect that quagga mussels would grow faster than zebra mussels in most years and that quagga mussels should increase to dominance, as commonly observed elsewhere [10, 15, 16, 39]. We also expect that quagga mussels should decline more than zebra mussels after round gobies increase in abundance resulting in a return of zebra mussels as the most abundant of the two dreissenid species when gobies are abundant.

### Study area

Onondaga Lake, New York (43°5’20” N, 76°12’30”W) is an 11.7 km^2^ meso-eutrophic lake with a mean depth of 10.9 m and a maximum depth of 20 m. For more than a century the lake has been the recipient of domestic and industrial wastewater from the Syracuse metropolitan area [40]. However, water quality in the lake has improved substantially during the past 25 years as a result of closures of several industries and improvements to the Syracuse Metropolitan Wastewater Treatment Plant (Metro) [41]. Several limnological parameters, including temperature, dissolved oxygen (DO), phosphorus, chlorophyll-a, and water clarity, as well as phytoplankton, zooplankton, and fish were monitored in this lake as part of an Ambient Monitoring Program run by Onondaga County Department of Water Environment Protection (OCDWEP) [38].

**Fig 1:**
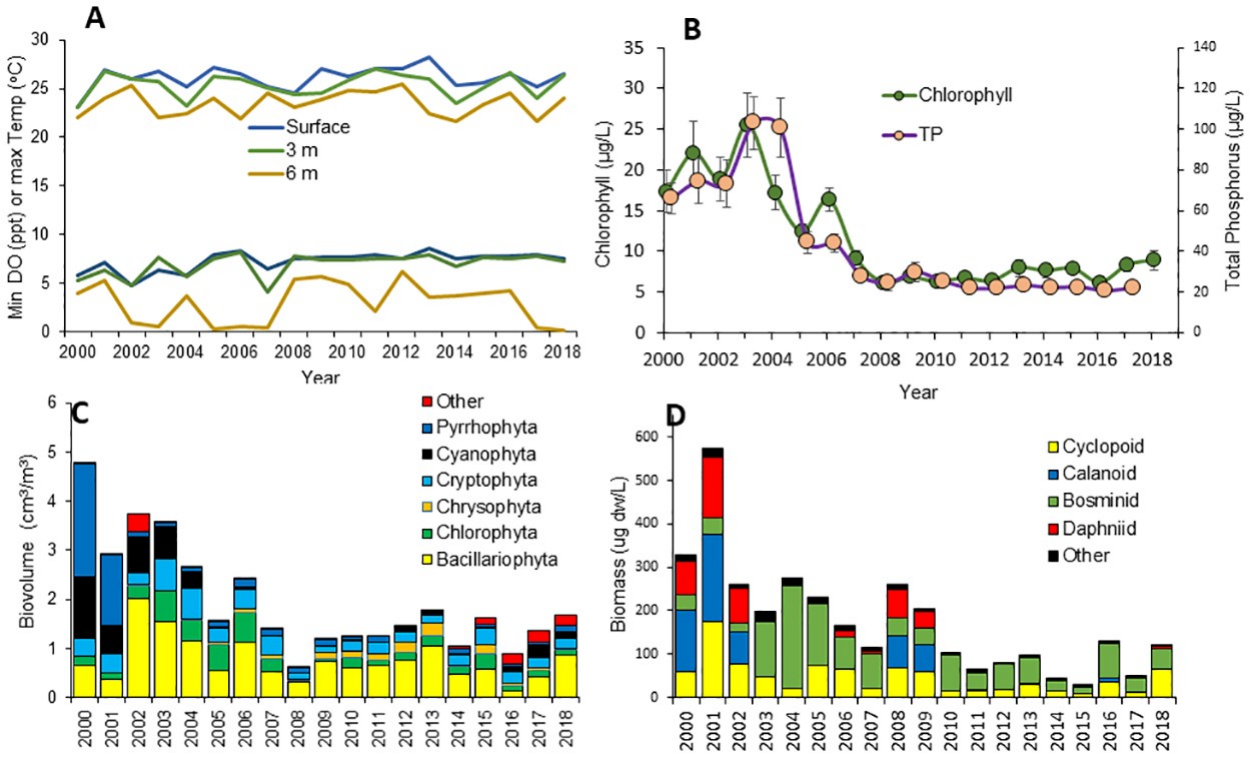
Time trends for limnological parameters in Onondaga Lake, 2005 to 2017. Values are based on bi-weekly samples at the south deep station. Panel A: maximum temperature (°C) and minimum oxygen concentrations (mg/L) measured at the surface, 3 and 6 m depths. The upper three lines are temperature and the bottom three lines oxygen. Panel B: total phosphorus (TP) and chlorophyll-a (April to November). Panel C: phytoplankton biovolume by major groups (April–October). Panel D: zooplankton dry biomass by major groups (April-October).

Although water quality improved over time, there was little additional change in the limnological parameters after 2007 [38]. Temperature and DO were measured bi-weekly at the surface, 3, 6, 9, 12, 15 and 18 m depth. Between year 2000 and 2018, maximum epilimnetic summer temperature ranged from 24.5 to 28.2 °C (Fig 1A), which is within the tolerance range of both mussel species [22]. Anoxic conditions in bottom waters started between the end of June and mid-July and continued to the fall overturn. In all years since 2000, water at 3 m depth remained oxygenated (DO>4 mg/L) throughout the year whereas DO at 6 m declined to less than 1 mg/L in some years (in 2002, 2003, 2005, 2006, 2007, 2017, 2018, Fig 1A). Annual average values for epilimnetic total phosphorus (TP) declined dramatically from 2000 to 2006, then remained in the range of 20–30 μg/L from 2007 to 2018 (Fig 1B). The time trends in chlorophyll-a concentrations were very similar to TP and remained between 6 to 10 μg/L from 2007 to 2018 (Fig 1B). Average annual Secchi disk transparency varied between 1.6 and 3.7 m, with no significant time trends. These trophic level indicators classify this lake as meso-eutrophic [42]. Annual average phytoplankton biovolume ranged from 0.5 to 2.0 cm^3^/m^3^ with diatoms the largest group followed by cryptophytes, chlorophytes and chrysophytes (Fig 1C). Zooplankton (Fig 1D) consisted of the common copepods and cladocerans of the region and was dominated by cyclopoid copepods and bosminids in years with abundant alewife (*Alosa pseudoharengus*), and by dahpniids and calanoid copepods in years with few alewife [43]. Change-point analyses [44] for the time period 2000–2018 show significant change points in years 2002 (total zooplankton), 2005 (TP and phytoplankton biovolume), and 2007 (chlorophyll), but not thereafter.

Both mussel species were reported from the outlet of Onondaga Lake in 1991, 3 years after they were documented as present in Lake Erie [45]. However, quagga mussels represented less than 1% of the mussels inspected in 1991 and although Mills et al. confirmed their presence in the spring of 1992, they could not find quagga mussels again in the fall of 1992 [6]. Both species of dreissenids remained rare in Onondaga Lake proper up to and including 1997 when reported densities were < 1 m^-2^ [46].

## Methods

**Fig 2:**
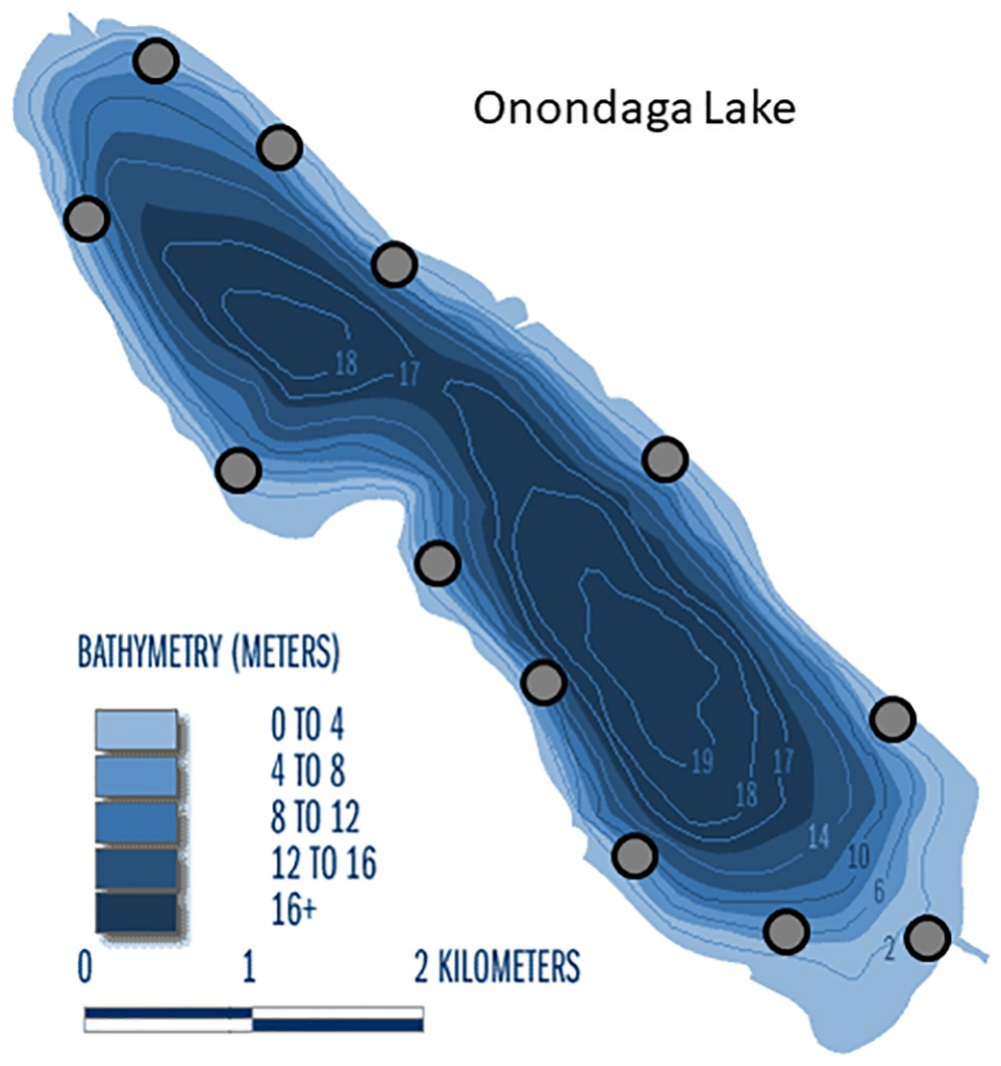
Onondaga Lake mussel sampling sites. Three (2005–2010, depth 0–4.5 m), four (2011–2012, depths 0–6 m), five (2013, depths 0–7.5 m) or six (2014, depths 0–9 m) samples were collected at each site with one sample collected per 1.5 m depth layer.

Mussels were sampled each year at depths 0–4.5 m at 12 sites around the lake from 2005 to 2018 (Fig 2) using ponar grabs (area 0.027 m^2^) by OCDWEP staff between October 8 and October 25. Ponar grabs were effective in Onondaga Lake because the substrate at all sites was basically the same (calcium carbonate enriched sand, silt, and organic material). At each site, one sample was collected from each of three depths 0–1.5 m, 1.5–3 m, and 3–4.5 m resulting in 12 clusters (= sites) of 3 grabs. This design was chosen to maximize variability within each site, as recommended in sampling design using cluster sampling [47]. Sampling prior to 2005 in Onondaga Lake [46, 48] and from nearby Oneida Lake [12] confirmed that bottom depth is an important gradient for mussel density, thus sampling across the depth gradient within each site is preferable to random selection of samples within each site [47]. The depths sampled were expanded to include a ponar grab at 4.5–6 m in 2011–2018, at 6–7.5 m in 2011–2018, and at 7.5–9 m in 2014–2018, as a response to improving oxygen conditions in the lake. We present time trends from 2005 to 2018 in water depth 0–4.5 m (depths sampled all years), and time trends from 2011–2018 in water depths 0–6 m (depths sampled since 2011). Samples were sieved in the field and processed in the laboratory. Up to 100–150 mussels that were alive at collection were measured in each sample to the nearest 0.1 mm (maximum shell length). When subsampled (samples with > 100 mussels), the weight of a random subsample of ~100 mussels and the weight of the total sample were measured to expand the numbers counted in the subsample to the whole sample. Total wet weight of the sample was measured to the nearest 1 g. Shell-on dry weight (SODW) was calculated from the lengths of each mussel measured using species-specific regressions from nearby Oneida Lake [12]:

$$\text{Quagga}\;\text{mussels}:{\text{log}}_{\text{e}}\left(\text{SODW}\right)=2.766*{\text{log}}_{\text{e}}\left(\text{SL}\right)-9.472$$ (1)

$$\text{Zebra}\;\text{mussels}:{\text{log}}_{\text{e}}\left(\text{SODW}\right)=2.864*{\text{log}}_{\text{e}}\left(\text{SL}\right)-9.622$$ (2)

where SODW is in g and SL is maximum shell length in mm. These calculated values were highly correlated with measured wet biomass in Onondaga Lake with no significant effect of mussel species, bottom depth, or year. Calculated SODW was 36.8% of measured shell-on wet weight (SODW (g) = 0.368 (SE 0.001) * wet weight (g), R^2^ = 0.99, N = 1322, P<0.0001). In nearby Oneida Lake, SODW was 35.3% of wet weight for zebra mussels and 33.9% of wet weight for quagga mussels with both SODW and shell-on wet weight measured on individual mussels [32]. We chose to analyze the calculated SODW values because small samples were not always weighed.

Round goby abundance was indexed with a beach seine at 15 sites in August and September each year. Each haul with the 15 m long, 1.2 m high beach seine covered an area of 116 m^2^. In all years, at least 2 surveys of these 15 sites were conducted and the numbers caught were expressed as a catch per seine haul. Round goby was also assessed with electrofishing at 12 transects in September. Electrofishing transects followed the shoreline in water depths of 1 to 2 m. Due to the large number of gobies encountered when electrofishing, only a portion of the observed gobies were captured and the number encountered along a transect but not captured was estimated by the operators. Electrofishing effort was standardized by power-on time (at output voltage 340 V) and given as the number of fish encountered per unit of power-on time (as per New York State Department of Environmental Conservation Fisheries Sampling Manual [49]). Time series from different fishing gear cannot be combined without standardization because catchability in different gear can be very different [50]. Therefore we standardized each catch per unit effort (CPUE) data series by dividing by the average annual CPUE in 2011–2018 for each gear, thereby making the CPUE relative to the average CPUE in 2011–2018 in each gear. This is a common method for comparing catches in different fishing gear [51]. We used the average of this normalized CPUE in seines and electrofishing as our index goby abundance.

To investigate the effect on mussels of the arrival of round goby, we tested for declines in density and biomass in water depth 0–6 m from 2011 to 2018 of (1) zebra mussel alone, (2) quagga mussel alone, and (3) both species combined. We averaged density and biomass from four (0–6 m depths) ponar samples to obtain an average per site. Standard errors in the figures were calculated using un-transformed values. Benthic animals are often aggregated making transformations of density values necessary [52]. Here we used fourth-root transformations for density and biomass which Strayer et al. [15] found appropriate for the dreissenid data series they analyzed, including the Onondaga Lake data up to 2015. Shell length and proportion quagga mussel were not transformed and standard errors were based on site values. We then tested for a time trend in the fourth-root transformed density and biomass data and for time trends in the proportion of quagga mussels using a mixed-model ANOVA with site as a random effect and year as a continuous fixed effect. Using site as a random effect accounts for consistent differences among sites. To test for difference in mussel length we used a paired t-test comparing mean and median lengths paired by year. For this test, mean lengths were first calculated from the measured mussels at each site, and then we calculated the average and standard errors of these site-specific mean lengths for all sites with more than 10 mussels measured (many sites had 100s of mussels measured). Median lengths were obtained from all measured mussels in a given year by species. Statistical analyses were done with Jmp ^®^ Pro 12.1 [53].

All sampling was done by OCWEP under collecting permits and guidelines obtained from New York State Department of Environmental Conservation.

## Results

**Fig 3:**
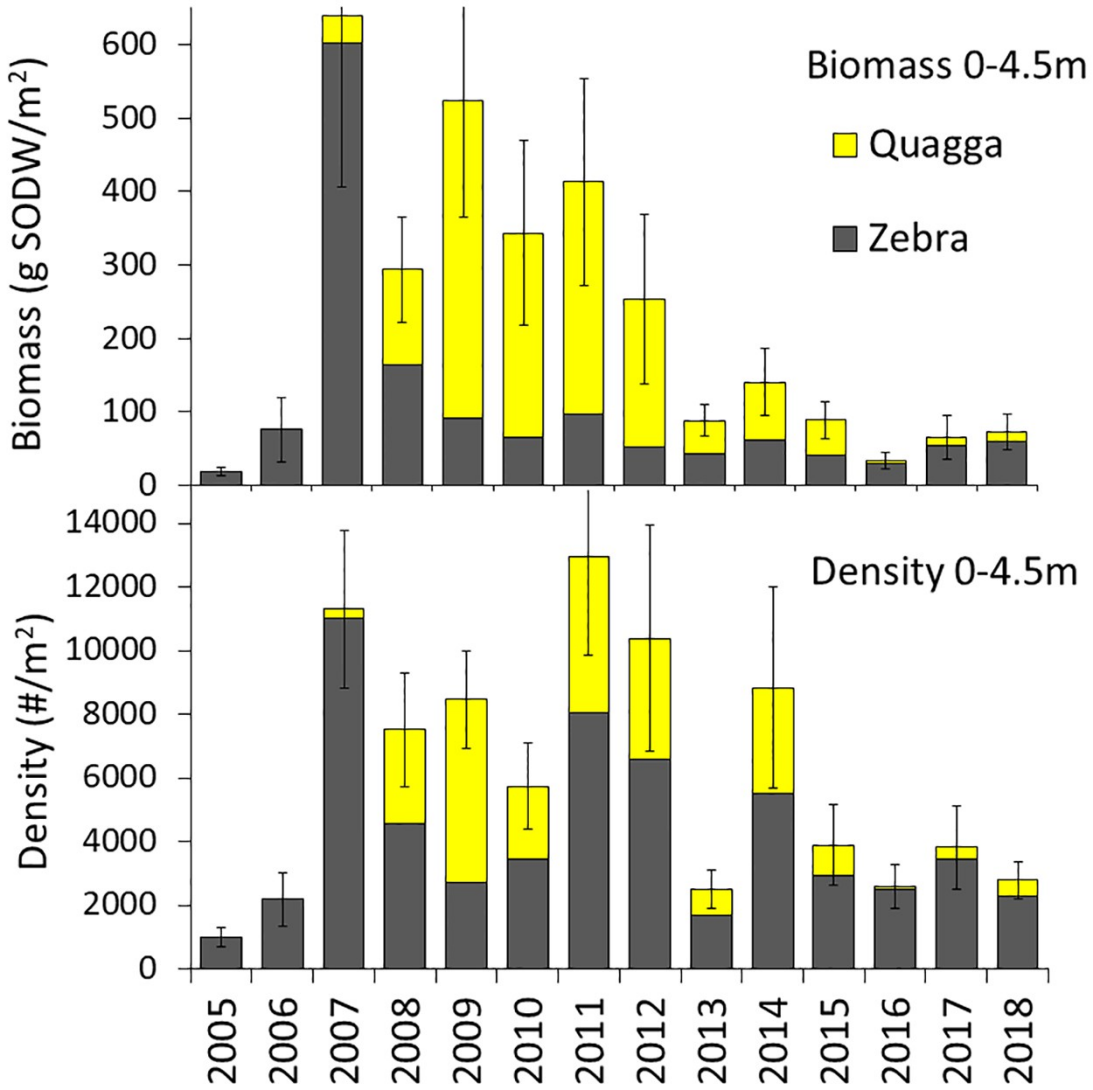
Development of the zebra and quagga mussel populations (biomass and density) in Onondaga Lake from 2005 to 2018. Depths 0–4.5 m were included. SODW is shell-on dry weight. Values are arithmetic means of site values. Bars represent ± 1 SE calculated using site averages of the sum of both species.

**Fig 4:**
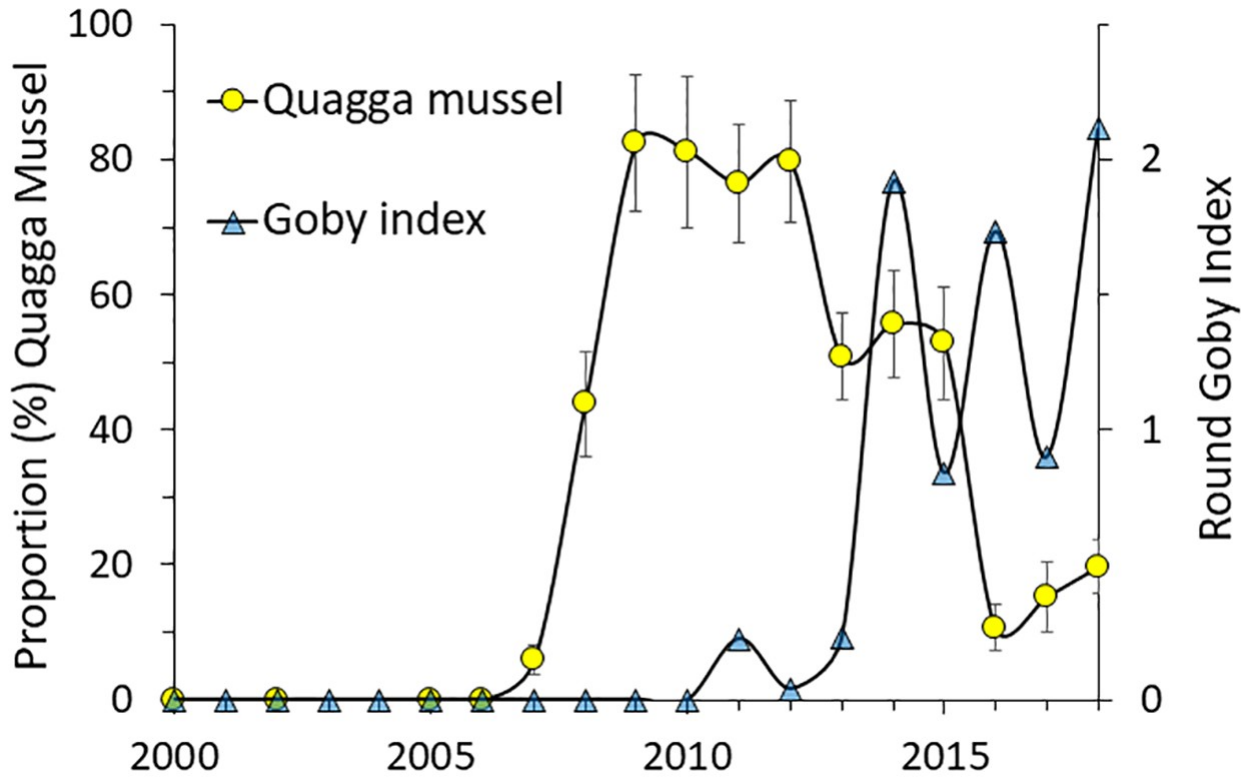
Proportion of the mussel biomass consisting of quagga mussels on bottom depths 0–4.5 m and an index of round goby abundance 2000–2018. The yellow circles are the proportion of quagga mussels (%), bars are ± 1 SE based on sites. Quagga mussel proportions for 2000 are from Spada et al. [46] and for 2002 are from a OCDWEP report [48]. Blue triangles represent the goby index calculated from beach seine and electrofishing surveys (see Methods).

Density and biomass (SODW, in parentheses) of zebra mussels on bottoms 0–4.5 m increased rapidly from 1,000/m^2^ (18 g/m^2^) in 2005 to 2,012/m^2^ (76 g/m^2^) in 2006 and 10,000/m^2^ (600 g/m^2^) in 2007. Zebra mussels then declined to between 1,655 and 7,705/m^2^ (42–165 g/m^2^) in 2008–2018 (Fig 3). Quagga mussels were not detected in 2005 and 2006 and present in low numbers in 2007 (294/m^2^, 38 g/m^2^). Density and biomass of quagga mussels increased to a peak of 5,721/m^2^ (432 g/m^2^) in 2009 and then declined to between 798 and 4,854/m^2^ (45–316 g/m^2^) in 2010–2014. Density and biomass continued to decline to between 71 and 513/m^2^ (4–14 g/m^2^) in 2016–2018 (Fig 3). The relative numeric abundance of quagga mussels increased from 3% to 67% from 2007 to 2009 and then decreased to 32–40% in 2010–2014 and 3–18% in 2016–2018. The proportion of the total dreissenid biomass (in 0–4.5 m depth) consisting of quagga mussels increased from 6% in 2007 to 76–84% in 2009–2012 and then decreased to 10–20% in 2016–2018 (Fig 4).

There were differences in density and biomass with depth with higher proportions of quagga mussels in deeper samples. Therefore, the addition of samples in 4.5–6 m from 2011 to 2018 increased the proportion of quagga mussels compared to values in 0–4.5 m depth shown in Fig 3. For example, in 2011–2012, the proportion of quagga mussel by biomass was 78% in depths 0–4.5 m and 97% in depth 4.5–6 m in 2011–2012. But even in samples collected in 4.5–6 m, the proportion of quagga mussels declined to 13% in 2017 and 54% in 2018. Mussels deeper than 6 m contributed on average 12% of the total biomass when such depths where sampled (range 2–30%, 2013–2018). No mussels were caught in 9–10.5 m samples in 2015, the only year such deeper bottoms were sampled.

**Fig 5:**
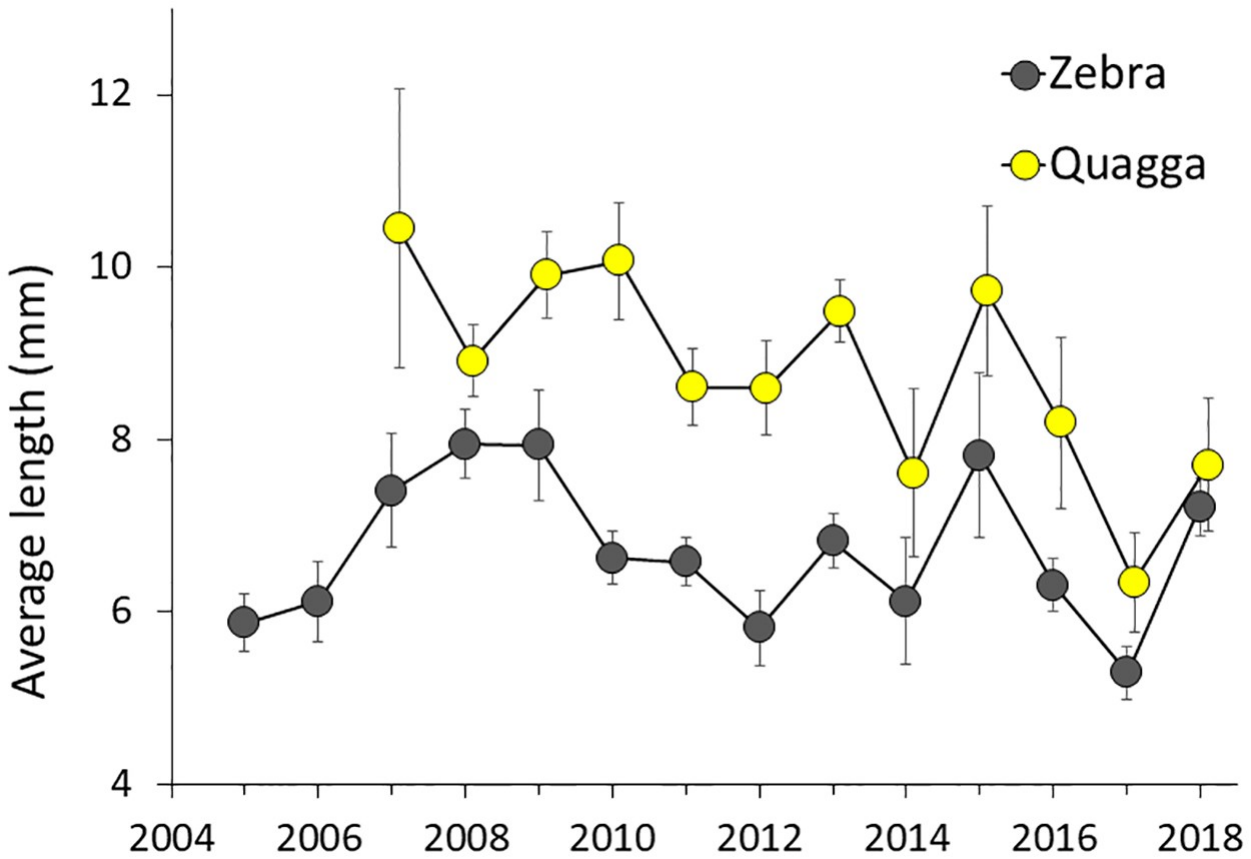
Average length of mussels in 0–6 m bottom depth in Onondaga Lake 2005–2018. Length is the maximum shell length. Bars are ± 1 SE calculated from the average lengths at the 12 sampled sites. Depths sampled: 0–4.5 m in 2005–2010 and 0–6 m in 2011–2018.

Mean length of measured quagga mussels (range among years 6.3–10.4 mm) was greater than the mean length of zebra mussels (Fig 5, range 5.3–7.9 mm) in all years. This difference was highly significant using a paired t-test with data points paired by year (P<0.0001, df = 11). Median lengths of all mussels from 0–6 m depth measured a given year gave the same results (median length range among years 5.3–14.7 mm for quagga mussel and 4.7–8.0 mm for zebra mussel, paired t-test, P = 0.0009, df = 11). Zebra mussels larger than 12 mm were uncommon in all years (2–19% of measured zebra mussels) whereas quagga mussels larger than 12 mm were more common (8–75% of measured quagga mussels). Mussels larger than 25 mm were rarely observed (17 quagga and 13 zebra mussels out of 34,534 individuals measured 2005–2018). In most years, the lengths distributions were unimodal.

**Fig 6:**
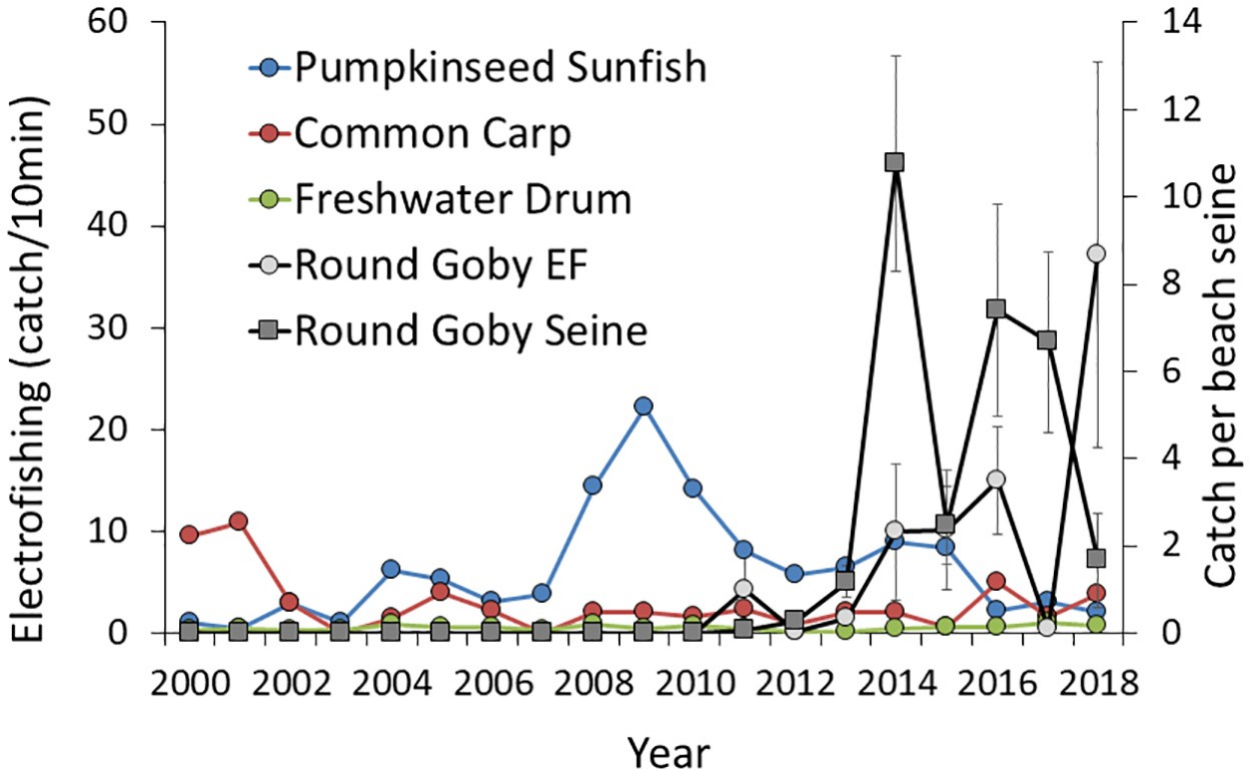
Abundance of molluscivorous fish in Onondaga Lake from 2005 to 2018. Data points represent catch per seine for beach seines and catch for 10 minutes of power-on time for electrofishing. Error bars are included for round goby (± 1 SE).

Round goby were first detected in Onondaga Lake in 2010. Goby densities increased from 2011 to 2013 in both beach seine surveys and electrofishing surveys and stayed abundant through 2018. Seine surveys may be the better index since all gobies caught were counted compared to electrofishing surveys where the number of gobies were estimated by the operators when abundance was high. However, with the exception of electrofishing in year 2017 when operators did not record estimates of gobies encountered but not captured during electrofishing, the pattern of increase from 2011 to 2013 and continued high abundance through 2018 is present in both gear (Fig 6). Other fish species were also sampled as part of the electrofishing surveys. Known molluscivores such as freshwater drum (*Aplodinotus grunniens*), common carp (*Cyprinus carpio*), and pumpkinseed sunfish (*Lepomis gibbosus*) show no significant time trends 2000–2018 (linear regression, all P>0.10) or 2005–2018 (all P>0.29, Fig 6) although pumpkinseed declined significantly from a peak CPUE in 2009 to 2018 (P<0.003). A decline in this predator is not consistent with a significant predatory effect of pumpkinseed on mussels that also declined during this time period.

**Fig 7:**
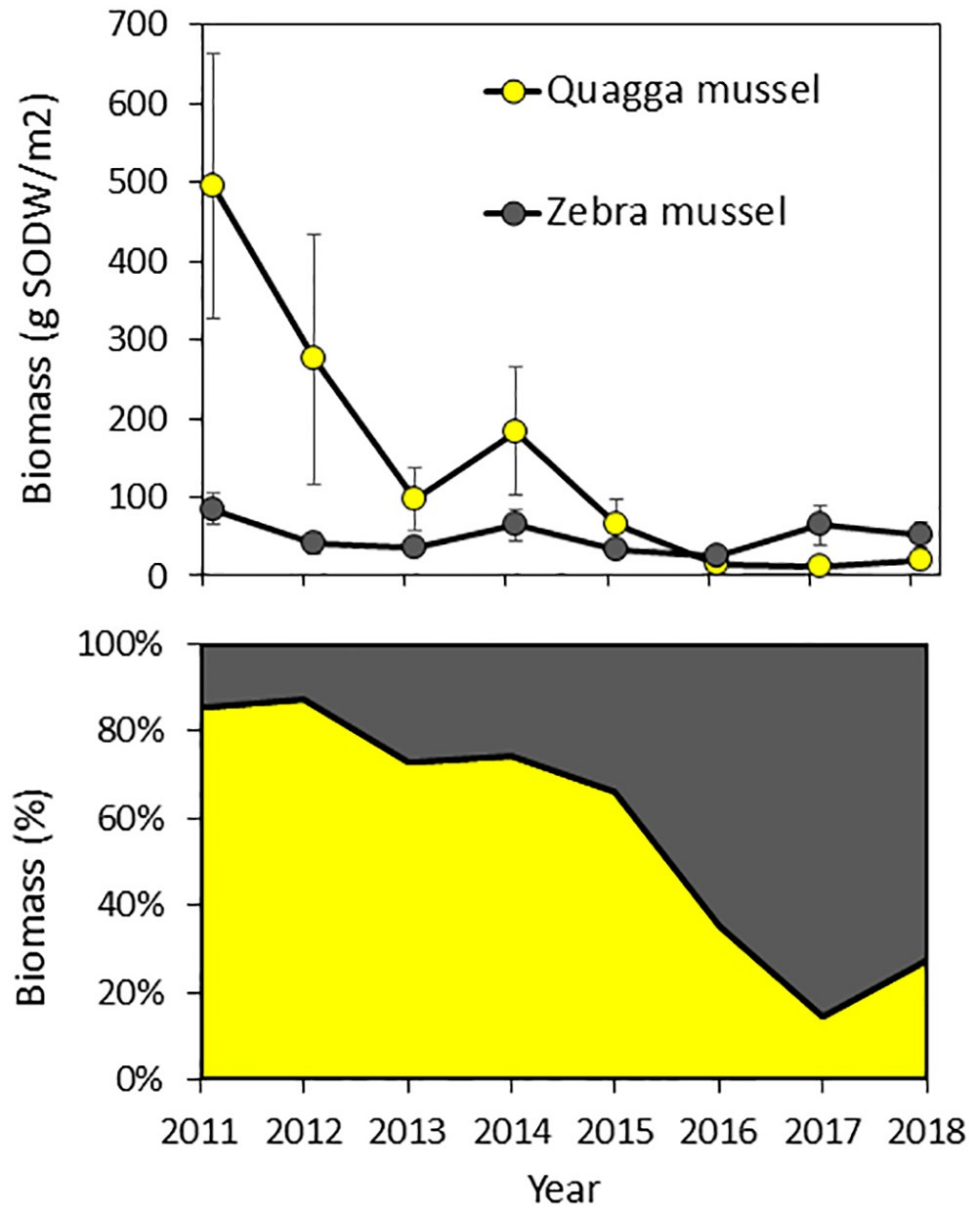
Biomass of zebra and quagga mussels in the 0–6 m bottom depths in Onondaga Lake from 2011 to 2018. Panel A: biomass with error bars indicating ± 1 SE. Panel B: the change in proportion by biomass of the two species.

The effects of the increase in round goby on mussel density and biomass was tested using the years 2011 to 2018; years sampled with 4 ponar grabs at each site collected between 0 and 6 m. Average density and biomass (SODW, in parenthesis) of both species in 0–6 m declined from 13,000/m^2^ (580 g/m^2^) in 2011 to 2,800/m^2^ (72 g/m^2^) by 2018 (Fig 7). The declines in density (year effect P = 0.0064) and biomass (year effect P = 0.0012) were both highly significant. Most of that decrease was due to a highly significant decrease in quagga mussels, as this species declined from 4,900/m^2^ (490 g SODW/m^2^) in 2011 to 510/m^2^ (20 g/m^2^) in 2018 (P<0.0001 for both density and biomass). Zebra mussels did not decline significantly during this time period (average density 4,123/m^2^, year effect P = 0.133, average biomass 50 g/m^2^, year effect P = 0.60). The proportion of quagga mussels also declined significantly both by biomass (P<0.0001, Fig 7) and density (P<0.0001).

## Discussion

The development of the dreissenid populations in Onondaga Lake up to 2011 was consistent with observations elsewhere [15]. This included the timing of peak abundance of both species [10, 12], the rate of the displacement of zebra mussels by quagga mussels [12, 16] and the higher growth rate of quagga mussels compared to zebra mussels [10, 11, 15, 54]. However, a return to zebra mussel as the most abundant of the two species, as observed between 2011 and 2018 has rarely been documented.

Both the initial increase of quagga mussels and the subsequent decline after the arrival of round goby in 2010 are consistent with the trade-off hypothesis suggested by Naddafi and Rudstam in 2014 [33]. They found that quagga mussels grew better than zebra mussels in the presence of predator cues as zebra mussels then invested more in anti-predator defenses. If this higher investment in anti-predator defense does not result in higher survival, as may be the case in newly invaded systems without mussel specialist predators, quagga mussels should dominate. This is a common observation in many newly invaded lakes and reservoirs including Onondaga Lake [15]. The trade-off hypothesis is also consistent with the larger size of quagga mussels in all years when they co-occurred in Onondaga Lake. More interesting, perhaps, is that the trade-off hypothesis also predicts a return to zebra mussels as the most abundant of the two dreissenids if predation rates on mussels increase and investment in anti-predator defenses therefore becomes more advantageous. This was observed in Onondaga Lake. After 2011, quagga mussels declined whereas zebra mussels did not, resulting in a return to zebra mussels as the most abundant mussel species from 2016 onwards. This decline occurred as the round goby, a known mussel specialist, became abundant.

The timing and magnitude of peak abundance of both species in Onondaga Lake was comparable to observations elsewhere. Peak density of zebra mussels typically occurs earlier after colonization (2.5 years on average) than peak density of quagga mussels (12.2 years after colonization, [10]). Zebra mussels were reported from the outlet from Onondaga Lake in 1991 [45]. However, the abundance of mussels remained low in the lake (<1 m^-2^) until 1999 when veliger counts increased and large number of 4–6 mm zebra mussels were found on trap nets [46]. Spada et al. [46] reported densities reaching 1,200 to 22,200 m^-2^ by year 2000, and most mussels between 5 and 15 mm shell length. They attributed this increase to improvements to the Metro sewage treatment plant after 1998, in particular to the reduction of ammonia as freshwater mollusks are sensitive to ammonia [55]. If water quality suppressed mussels before 1998, zebra mussels would have reached high densities 2 years after the lake became conducive to dreissenids, similar to the time lag between arrival and peak abundance observed elsewhere [9]. Quagga mussels were reported in very low numbers from the outlet of Onondaga Lake in 1991 [45] and in spring of 1992 but were not found in the fall of 1992 [6, 46]. Stewart [56] documented an eastward progression of quagga mussels along the Erie Canal from 1998 to 2009. At the outlet to Onondaga Lake, quagga mussels were not found in 1998, 1999, 2000 or 2002, but dominated in 2009 [56]. Similarly, no quagga mussels were reported from the 2000 survey in the lake proper [46], but a few quagga mussels were found in a 2002 survey [48]. After 2007, quagga mussels increased rapidly and the species went from a minor component of the dreissenid population in 2007 to having a higher biomass than zebra mussels in 2009, 2 years later. This rate of increase of quagga mussels is similar to the rate of increase observed in European lakes (26% per year, [16]) and in nearby Oneida Lake [12]. Peak quagga mussel abundance in 2009 is 11 years since 1998 when presumably also quagga mussels could have increased in the lake if present, or 7 years since 2002, when they were first reported from the lake proper. This is within the range of observations elsewhere for the time to peak abundance of quagga mussels in lakes initially dominated by zebra mussels (6–19 years, [9]). Peak densities of dreissenids in Onondaga Lake (> 10,000 /m^2^ in 0–6 m) were also comparable to observations elsewhere [15, 57, 58]. Lake-wide densities would be lower because the 70% of the lake bottom that is below 6 m depth can be anoxic during the summer and had few dreissenid mussels when those depths were sampled. Quagga mussels were larger than zebra mussels in all years with data on both species. Comparisons of growth rates of the two species under similar conditions are relatively rare; most studies report higher growth of quagga mussels [12, 15, 19, 59], but see [60].

Several hypotheses have been proposed for the mechanisms behind the initial displacement of zebra mussels by quagga mussels including the quagga mussel’s ability to grow and reproduce at cold temperature and at lower food concentrations [9, 10, 22]. Because quagga mussels did become dominant in Onondaga Lake, a lake with relatively high levels of edible algae and without habitable cold water bottoms due to summer anoxia, cold water and low food concentrations are not necessary for quagga mussels to dominate. However, the trade-off hypothesis predicts a dominance of quagga mussels also in productive lakes, like Onondaga Lake, if predation rates are low. Quagga mussels were larger than zebra mussels in all years, also consistent with the effect of predator cues decreasing zebra mussel growth more than quagga mussel growth. We note that other hypotheses, such as lower metabolic rate and higher growth efficiency of quagga mussels may also be important (reviewed by Karatayev et al. [10], but these mechanism may be the results of lower investment in anti-predatory defenses and are not in conflict with the trade-off hypothesis.

Predation rates on mussels should increase with the invasion of round goby, a dreissenid specialist [32, 61]. Round goby can consume more mussels per unit time than crayfish and native molluscivorous fish, such as pumpkinseed sunfish [62]. Round goby arrived to Onondaga Lake in 2010, increased in abundance up to 2013 and has remained abundant through 2018. Total dreissenid mussel abundance did decline from 2011 to 2018 primarily because of declines in quagga mussels. The result was a return to zebra mussel as the most abundant of the two species by 2016, with the largest decline in quagga mussels after 2013 when round goby became abundant. Zebra mussel continued as the more abundant species through the end of our study in 2018, consistent with continued high round goby densities in the lake. Elsewhere, predation has been suggested to be an important source of dreissenid mortality; and fish, larger crustaceans, and diving ducks are all considered important predators on dreissenids in Europe and North America [63–71].

We did consider other possible explanations for both the initial displacement of zebra mussels by quagga mussels, and the subsequent decline of quagga mussels and return of zebra mussel as the most abundant species. Change point analysis of the limnological time series indicate that significant changes occurred in the time period 2002 to 2007, with less change after 2007 –the period of the largest changes in the two mussel populations. Measurements of temperature and dissolved oxygen were within the expected tolerance of both dreissenid mussels with the exception of low oxygen concentration at 6 m in some years. Low oxygen at 6 m in 2017 and 2018 could have contributed to fewer deep quagga mussels those years [72], but oxygen was sufficient at 3 m in all years, and quagga mussels decreased from ~ 50% in 2013–2015 to 5–27% in 2016–2018 also in 1.5–3 m depths. Other predators than round goby could also be important, but fish species known to feed on mussels did either not change in abundance or declined with the decline in mussels. Both crayfish and diving ducks are known predators on mussels [63], and diving ducks do congregate on the lake during spring and fall migrations. Although we cannot rule out a surge in ducks or crayfish from 2011 to 2018, at least crayfish also prefer quagga mussels over zebra mussels [33] and if they did increase would contribute similarly to round goby to the return of zebra mussels. There are of course other possibilities, such as an increase in diseases and parasites [73, 74] that we did not evaluate. However, we consider the most likely cause for the decline in quagga mussels and total dreissenids to be the arrival and subsequent increase of round goby. Note that zebra mussels did not decline significantly with the increase in round goby, and zebra mussels therefore returned to being the most abundant of the two dreissenids.

Although there are many examples of the increase in dominance of quagga mussels, there is only limited evidence for a reversal to zebra mussels as the most abundant of the two species. None of the 42 longer-term (>10 year) data series on adult dreissenid mussels from Europe and North America analyzed by Strayer et al. [15] showed a differential decline of quagga mussels, and there was no general decline in the combined dreissenid mussels with time since invasion. But only four of these 42 data sets included more than 10 years of annual data on adult mussels from systems with both quagga and zebra mussels (Oneida Lake [12], Hudson River [75], Lake Balaton [54], and Onondaga Lake—this study). Interestingly, in the Hudson River, quagga mussels have remained subdominant for decades, perhaps due to higher predation rates in the river [75]. There are also studies that were not included in the Strayer et al. data set that suggest a link between predator abundance and mussel species dominance. Zhulidov et al. [76, 77] did observe a shift from quagga mussel dominance to zebra mussel dominance in the lower Don River system, and speculated that selective predation on quagga mussels by roach (*Rutilus rutilus*) adapting to mussel feeding could explain the return of zebra mussel dominance. In addition, twelve years of annual data from lakes Erie, Ontario, Michigan and Huron have recently been published [11, 78] and the data from western Lake Erie where round goby is abundant (but not from the deeper lakes Ontario, Michigan and Huron) show coexistence of the two dreissenid species. The two species also continue to coexist in the shallow water of Oneida Lake [12, 79].

A decline in the density of an invasive species following an initial peak in abundance may be expected as the invaded community adapts to the presence of the new species [80]. This may also be the case for dreissenid mussels [10], although the evidence for such a decline is stronger for density than biomass and is not always observed [15]. Even so, when declines occur, they have a cause. The Onondaga Lake data supports increased predation as an important mechanisms contributing to such declines. Further, the trade-off hypothesis predicts both the quagga mussels dominance over zebra mussels also in productive systems and the disproportionate decline in quagga mussels following an increase in predation rates, such as expected after the round goby invasion in Onondaga Lake. If the trade-off hypothesis is correct, we predict that zebra mussels will continue to be the more abundant of the two species in Onondaga Lake as long as round goby remain abundant. Whether or not zebra mussel will increase as quagga mussel declines likely depend on the relative importance of increased predation mortality and increased population growth associated with decreased competition with declining quagga mussels. In Onondaga Lake and the years studied here, zebra mussels did not change significantly with the invasion of round gobies; elsewhere the results may be different. However, we expect that quagga mussels will continue to have a competitive advantage in low predation environments and in the cold oxygenated bottoms of deep lakes. Thus, the relative abundance of the two species should vary among lakes with deep oligotrophic lakes dominated by quagga mussels, shallow lakes with high predation pressure dominated by zebra mussels, and coexistence of both species in intermediate habitats.
